# A comparison of real-world data on adjuvant treatment in patients with stage III BRAF V600 mutated melanoma – Results of systematic literature research

**DOI:** 10.1016/j.ejca.2024.115160

**Published:** 2024-12-13

**Authors:** Teresa Amaral, Lena Nanz, Lina Maria Serna Higuita, Paolo Ascierto, Carola Berking, Eva Muñoz Couselo, Marco Donia, Reinhard Dummer, Ralf Gutzmer, Axel Haushild, Mathilde Jalving, Rebecca Lee, Paul Lorigan, Ivan Marquez-Rodas, Olivier Michelin, Paul Nathan, Caroline Robert, Dirk Schadendorf, Pawel Sobczuk, Lukas Flatz, Ulrike Leiter, Claus Garbe

**Affiliations:** a Center for Dermato-oncology, Department of Dermatology, Eberhard Karls University of Tübingen, 72076 Tübingen, Germany; b Cluster of Excellence iFIT (EXC 2180) “Image-Guided and Functionally Instructed Tumor Therapies”, Tübingen, Germany; c Clinical Epidemiology and Applied Biostatistics, Eberhard Karls University of Tübingen, 72076 Tübingen, Germany; d Melanoma, Cancer Immunotherapy and Development Therapeutics Unit, Instituto Nazionale Tumori IRCCS Fondazione Pascale, Napoli, Italy; e Department of Dermatology, Uniklinikum Erlangen, Friedrich-Alexander-Universität Erlangen-Nürnberg, Deutsches Zentrum Immuntherapie and Comprehensive Cancer Center Erlangen-European Metropolitan Area of Nuernberg, (CCC ER-EMN), Erlangen, Germany; f Vall d’Hebrón Universisty Hospital, Barcelona, Spain; Vall d’Hebrón Institute of Oncology (VHIO), Barcelona, Spain; g National Center for Cancer Immune Therapy, Department of Oncology, Copenhagen University Hospital, Herlev, Denmark; h University Hospital Zurich, Department of Dermatology, Switzerland; i Department of Dermatology, Johannes Wesling Medical Center Minden, Ruhr University Bochum, 32429 Minden, Germany; j Department of Dermatology, University Hospital (UKSH), Kiel, Germany; k Department of Medical Oncology, University Medical Centre Groningen, the Netherlands; l Faculty of Biology, Medicine and Health, The University of Manchester, Oxford Road, Manchester M13 9PL, UK; m Department of Medical Oncology, The Christie NHS Foundation Trust, Manchester M20 4BX, UK; n Medical Oncology Department, Hospital General Universitario Gregorio Marañon, Madrid, Spain; o Department of Oncology, Geneva University Hospital, Geneva, Switzerland; p Mount Vernon Cancer Center, Northwood, UK; q Department of Oncology, Institute Gustave Roussy and Paris-Saclay University, Villejiuf, France; r Department of Dermatology, Comprehensive Cancer Center (Westdeutsches Tumorzentrum), University Hospital Essen & National Center for Tumor Diseases (NCT-West), Campus Essen & Research Alliance Ruhr, Research Center One Health, University Duisburg-Essen, Essen, Germany; s Department of Soft Tissue/Bone Sarcoma and Melanoma, Maria Sklodowska-Curie National Research Institute of Oncology in Warsaw, 02-781 Warsaw, Poland

**Keywords:** Adjuvant therapy, Targeted therapy, Checkpoint inhibitors, Stage III, Relapse-free survival, Distant metastasis-free survival, Overall survival

## Abstract

**Background:**

Over the past decade, PD-1-based immune checkpoint inhibitors (ICI) and targeted therapies (TT) with BRAF and MEK inhibitors transformed melanoma treatment. Both are widely used in the adjuvant setting. However, for patients with a BRAF V600 mutation, the optimal adjuvant therapy remains unclear due to the lack of head-to-head comparison studies.

**Methods:**

We conducted a systematic review of real-world data on adjuvant therapy in stage III melanoma to determine the best option for patients with BRAF V600 mutations. Kaplan-Meier curves were generated for TT and ICI using Digitizelt software.

**Results:**

Nine publications with 3625 patients were included. TT showed better relapse-free survival (RFS) at 6, 12, 24, and 36 months than ICI. A similar trend was observed for distant metastasis-free survival (DMFS), with no apparent difference in overall survival.

**Conclusion:**

Real-world data suggest that adjuvant TT may be associated with better RFS and DMFS in stage III BRAF V600-mutated melanoma compared to ICI.

## 1 Introduction

Over the last decade, two important strategies have been developed for the systemic treatment of melanoma: PD-1 based immune checkpoint inhibition (ICI) and targeted therapy (TT) with BRAF and MEK in-hibitors. The latter is only applicable if a BRAF V600 mutation is present, with only a few exceptions. For the 40–50 % of patients with melanoma having this mutation, both ICI and TT can be offered. This raised the question of which therapy should be used in first line.

For patients with unresectable stage IV disease, clinical trials comparing ICI versus TT in first line were conducted, and the results reveal that patients receiving a combination of ICI in first line have a better outcome, with TT being given in second line. [1,2] In resected stage III melanoma, both therapeutic strategies including nivolumab or pembrolizumab (ICI) and dabrafenib plus trametinib (TT) have been approved for adjuvant treatment. [3–5] However, in this setting, there have been no comparative, head-to-head randomized clinical trials to date, and they are likewise not expected. On the other hand, a substantial body of real-world data on adjuvant treatment outcomes for patients with stage III melanoma is now available.

We conducted a systematic literature review of real-world data on adjuvant treatment for stage III melanoma. Our objective was to determine whether real-world data can address which adjuvant therapy offers the best survival outcomes, namely relapse-free survival (RFS), distant metastases free survival (DMFS) and overall survival (OS), for patients with a BRAF V600 mutation.

## 2 Methods

### 2.1 Search strategy and selection criteria

We searched PubMed from Jan 1, 2012, to Feb 29, 2024, with the following two algorithms: 1) “Melanoma [ti] AND adjuvant [ti] AND real world [ti]”; and 2) “Melanoma [ti] AND adjuvant [ti] AND real world”. We also searched relevant articles referenced by other publications and abstracts from clinical meetings held in this time period. All publications selected were available either as full text publications or as conference abstracts. Publications that did not include survival outcomes of TT or ICI in patients with BRAF V600 mutation were excluded. A summary of the publications included in the analysis is provided in Table 1.

### 2.2 Statistical analysis - Kaplan Meier after digitalization

The RFS survival data were obtained by estimating individual data by Kaplan Meier curves in the selected publications. The RFS probabilities at pre-specified time intervals were extracted from the published Kaplan-Meier curves using the software Digitizelt. [6,7] Digital sampling points were created from published Kaplan-Meier survival curves, and digital curves were constructed by linear interpolation between these points. Mean survival curves were then calculated as a weighted average of the survival curve from the studies included in our analysis. The weights applied were in proportion to the number of individuals stratified by therapy (ICI vs. TT). Weighted averaging was calculated pointwise at the sampling points t_k_ from all digitized Kaplan-Meier curves S_i_(t) using the following formula:

$$\hat{\text{S}}(\text{tk})=\frac{\sum \text{ni}\hat{\text{S}}\text{i}(\text{tk})}{\sum \text{ni}}$$

where: **Ŝ(t**_**k**_**):** represents the value at time point **(t**_**k**_**)** and **n**_**i**_ represents the initial sample size estimation. [6,7] In addition, Kaplan-Meier curves stratified by individual publication were created to evaluate their consistency with the weighted Kaplan-Meier analysis.

We refrained from statistical tests for evaluating differences in survival between TT and ICI, and limited ourselves to descriptive evaluations, as the composition of the populations in the various publications was quite different.

## 3 Results

### 3.1 Population included

Data from nine publications with real-world data were included in the analysis, comprising a total of 3625 patients. Median follow up ranged between 11 and 33 months.

**Fig. 1:**
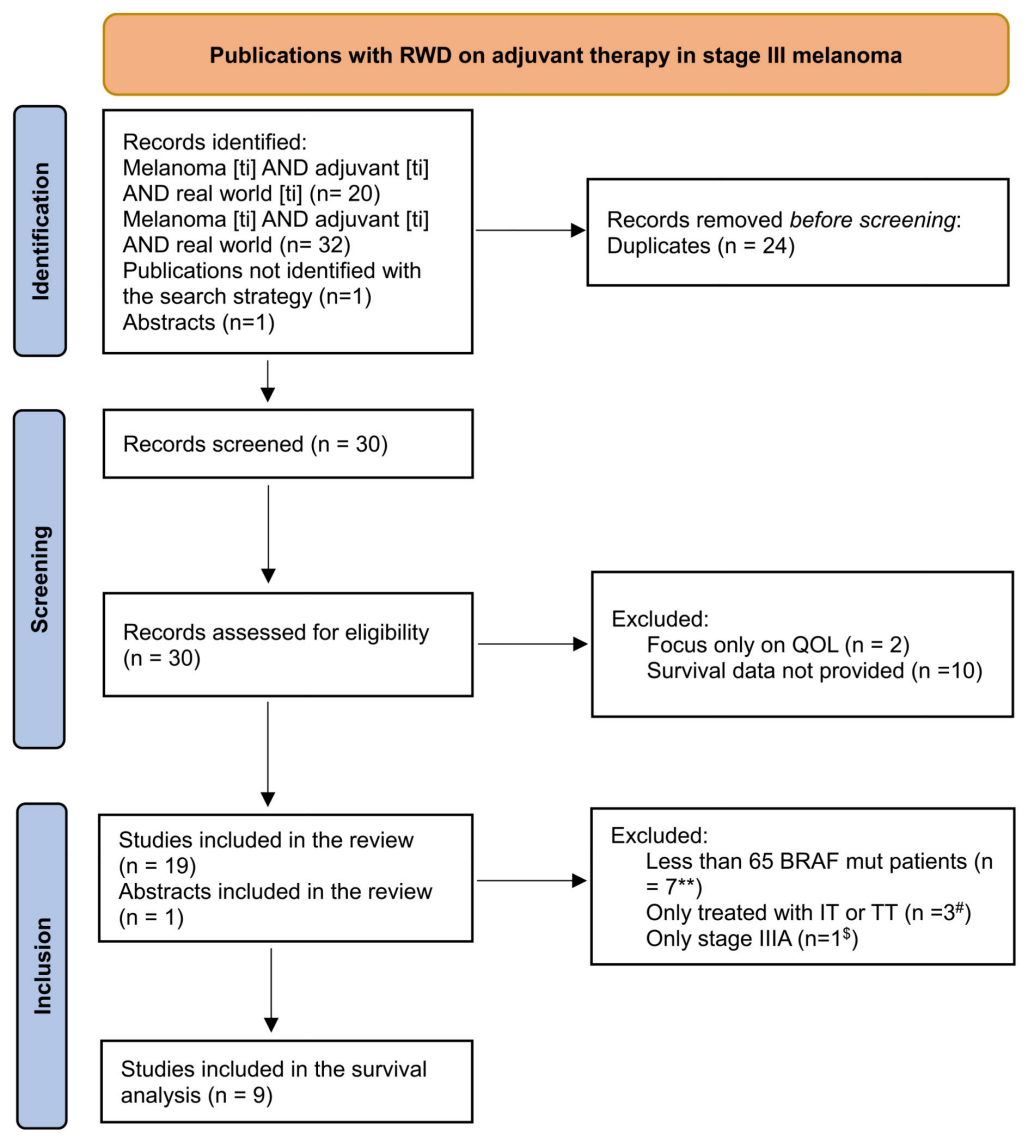
Consort diagram.

Fifty-seven percent of patients (n = 2071) had a BRAFV600 mutation. Fifty-six percent of all BRAF V600 mutated patients (n = 1164) were treated with TT therapy, and 44 % (n = 907) were treated with ICI. [8–16] Six out of nine publications reported data on the subtype of BRAF V600 mutation of the patients included, with the majority being BRAF V600E, as expected. More data on the subtype of BRAF V600 mutation is available in Table 1 and S1, for the publications that reported this information. The consort diagram can be found in Figure 1.

### 3.2 Analysis of survival outcomes

A total of 20 Kaplan Meier curves for RFS were extracted from the above-mentioned 9 publications (n = 3625 patients). Data on DMFS and OS was also extracted as follows: 5 Kaplan Meier curves for DMFS were extracted from 2 publications (n = 608 patients) and 8 Kaplan Meier curves for OS were extracted from 4 publications (n = 2559 patients).

After digitalization, the survival curves were grouped by treatment type, i.e., TT or ICI. Weighted averaging of RFS curves was performed within each therapy strategy group as described above and plotted separately for TT and ICI (Fig. 2A). Patients with BRAF V600 mutation treated with TT seem to have better RFS outcomes than patients treated with ICI. Weighted averaging of DMFS and OS curves was also performed within each therapy strategy group as described above and plotted separately for TT and ICI (Figs. 2B and 2C). We also observed a trend for better DMFS in patients with BRAF V600 mutation treated with TT, although data were reported in only two publications. In terms of OS benefit, there were no obvious differences between TT and ICI, but this conclusion is based on data from only four publications.

### 3.3 Survival with targeted therapy compared to immune checkpoint therapy

A key objective of this exploratory analysis was to compare the mean survival curves of patients receiving TT versus ICI.

Averaged survival proportions, specifically the RFS, DMFS and OS rates at 6, 12, 24, and 36 months for TT and ICI, were calculated and are available in Table 2. The RFS rates at the different time points indicated a higher numerical value for TT compared to ICI in patients with BRAF V600 mutation. The 6, 12, 24, and 36 months RFS rates were 95 % vs 82.3 %; 85.8 % vs 68.5 %; 67.4 % vs 54.6 % and 51.9 % vs 48.3 %, respectively for TT and ICI.

### 3.4 Survival with targeted therapy and immune checkpoint therapy

To further evaluate the concordance between the outcomes of TT and ICI among the different publications, we performed grouping of digitized Kaplan Meier RFS curves, side by side (Figures 3A and 3B). The results showed a high concordance between the different publications within each therapy.

## 4 Discussion

Our analysis of real-world data on the outcomes of adjuvant therapy in 3625 patients with stage III melanoma suggest that TT may provide better RFS benefit than ICI in patients with BRAF V600 mutation, and this is maintained at 1 and 2 years of follow-up (86 % vs 68 % and 67 % vs 55 %, respectively). The same trend was observed for DMFS, although the number of patients included was lower. There was no obvious difference in terms of OS between both therapies. Longer follow-up is needed to confirm these results.

Currently, based on the results from the NADINA and SWOG S1801 trials [17,18] a therapeutic option for patients with Stage III resectable macroscopic disease is 2 cycles of neoadjuvant ipilimumab plus nivolumab followed by complete surgical resection and adjuvant therapy according to pathological response and BRAF status, or 3 cycles of neoadjuvant pembrolizumab followed by complete surgical resection and 15 cycles of adjuvant pembrolizumab. Still, a significant percentage of patients present with microscopic stage III disease, only detected after complete resection (i.e., sentinel lymph node biopsy). Therefore, adjuvant TT for patients with BRAF V600 mutated melanoma and ICI for BRAF wild-type and BRAF V600 mutated melanoma can be considered. Additionally, the comparator arm in the NADINA study did not include adjuvant TT alone, therefore a direct comparison between neoadjuvant ICI and adjuvant targeted therapy cannot be made at this time point.

Recently, the first OS data in adjuvant stage III melanoma were reported in the COMBI-AD study using dabrafenib plus trametinib. It was shown that for the whole population of patients with BRAF V600-mutated melanoma there was no statistically significant OS benefit of adjuvant TT versus placebo (hazard ratio (HR) for death, 0.80; 95 % confidence interval [CI], 0.62 to 1.01; p = 0.06). [19] However, when BRAF V600 mutation types were analyzed separately, the HR for death for patients with BRAF V600E was 0.75 (95 % CI, 0.58 to 0.96) and for patients with BRAF V600K was 1.95 (95 % CI, 0.84 to 4.50). This means that patients with the more common BRAF V600E mutation experienced a significant improvement in OS with TT, whereas patients with the less frequent BRAF V600K mutation could have a survival disadvantage. However, this was a subgroup analysis, and the results should be interpreted cautiously. The primary endpoint of the COMBI-AD study was RFS which showed a clear benefit for patients receiving adjuvant TT compared to placebo; the median RFS was 93.1 months (95 % CI, 47.9 to not reached) for dabrafenib plus trametinib and 16.6 months (95 % CI, 12.7 to 22.1) for placebo (HR for relapse or death, 0.52; 95 % CI, 0.43 to 0.63). The absence of benefit in OS despite the RFS and DMFS benefit when using adjuvant therapy, also in the real-world setting, may be explained by the use of other systemic therapies, such as immune checkpoint inhibitors, at the time of relapse, which seem to be able to rescue patients in a more advanced, irresectable stage. Specifically in the COMBI-AD study, and for the safety population analysis, 97 of 213 patients receiving placebo were treated with PD-1 based therapies at any time after relapse. This relatively low number may be due to early relapses during the study when PD-1 based therapy was not available outside clinical trials. Also worth mentioning is that the COMBI-AD OS analysis was done with only 260 of the 597 events included in the initial statistical plan, which may have contributed to the absence of OS benefit seen.

The presence of BRAF V600 mutation is a prognostic factor. Indeed, patients with untreated BRAF V600 mutated melanoma have a worse prognosis than patients with BRAF wild-type tumors. [20] However, there seems to be no difference in terms of RFS or DMFS benefit in patients with BRAF V600 mutated compared to wild-type tumors treated with ICI, suggesting that patients with BRAF V600 mutated tumors may have a relative higher benefit. [21].

Currently, there are no OS data reported from the adjuvant trials investigating ICI therapy versus placebo in stage III. There is some uncertainty if the RFS/DMFS benefit in the adjuvant setting, similar to TT, will translate into OS benefit. Indeed, the first OS data from Keynote 054 initially planned to be reported in 2023 have now been postponed to Q4 of 2027. [22] Further studies on adjuvant ICI therapy in resected stage III and IV melanoma, namely Checkmate 238 and SWOG 1404 trials, showed a benefit in terms of RFS but not OS, although in both trials the comparator arms were active treatments, i.e., ipilimumab and α interferon or ipilimumab, respectively, instead of placebo. [23,24].

When discussing adjuvant therapy with patients with melanoma, the toxicity profile of the individual options must be considered. Adverse events associated with TT are generally transient and diminish or resolve with therapy pause, although they may recur when therapy is resumed. Permanent or irreversible toxicities may occur, but are rare. [25] On the other hand, toxicity associated with ICI, depending on the CTCAE grade, may require not only therapy pause but also immuno-suppression. Moreover, some toxicities associated with ICI, such as hypothyroidism, hypophysitis and diabetes mellitus, are irreversible and require lifelong hormone supplementation. [26].

Another aspect that needs to be discussed is the quality of life (QOL) reported in association with TT and ICI. In Keynote 054, a decrease in QOL was reported for patients receiving pembrolizumab compared to placebo, although this difference remained below the clinical relevance threshold. [27] Data from real-world show that adjuvant ICI seems to be associated with a significant decline of QOL at 12 months compared to baseline. [28] Rogiers et al. reported that during ICI adjuvant treatment (at 9 months), the percentage of patients with clinically significant fatigue and cognitive impairment increased compared to baseline. [29] After treatment ended (at 18 months), reports of clinically significant fatigue decreased, but there was an increase in the percentage of patients with clinically significant emotional, cognitive, and social impairment compared to during treatment. Pedersen at al. also reported that adjuvant nivolumab may affect QOL at least temporarily. [30] As for adjuvant TT, the QOL analysis of patients receiving dabrafenib and trametinib in the COMBI-AD study showed that TT did not adversely affect health-related QOL during treatment or in long-term follow-up, in the absence of disease-related symptoms. [31] Important to consider that there are no data specifically looking into the QoL of patients who relapsed on placebo therapy or comparing head-to-head QoL of adjuvant targeted and immunotherapy.

Another aspect to consider when selecting the adjuvant therapy regimen may be the potential development of resistance to systemic therapy in more advanced stages. So far, the data available is scarce. The benefit may be different depending on the type of relapse, i.e., relapse on or off adjuvant therapy. Patients who relapsed after adjuvant TT seem to benefit from subsequent anti-PD-1 based therapy with outcomes similar to first line or therapy naïve stage IV melanoma, i.e., TT did not impacted response to ICI. [32] On the other hand, patients with recurrence on PD-1 adjuvant therapy seem to have marginal benefit from PD-1 monotherapy but may benefit from ipilimumab, alone or in combination with PD-1, and TT. [33] Still, in a real-world setting comparing patients receiving and not receiving adjuvant therapy, there was no OS difference, suggesting that the use of systemic therapies may be able to rescue patients who have a recurrence. [34] More data is necessary here, before drawing definite conclusions.

Our analysis has limitations: 1) This is an indirect comparison using real-world data that is not documented as detailed as in a clinical trial. Capture of RFS events depends on the radiology evaluation schedule, which diverges among countries and centers, contrary to the standardized evaluation in clinical trials. 2) The criteria for including the publications in our analysis were similar (adjuvant therapy in stage III melanoma), but the criteria for including the patients in the different analysis differ between publications. A potential selection bias could be present in this analysis, as some patients’ basal characteristics (i.e. Breslow index, ulceration, number of involved nodes, localization of primary tumor, LDH level, age, lymphocyte counts, performance status, etc.) may be imbalanced in the treatment groups. 3) Patients treated with ICI were both BRAF wild-type and BRAF V600 mutated, as without access to the raw data from the different publications it was not possible to distinguish these populations when extracting the data from the ICI survival curves. 4) Survival data for TT in patients with BRAF V600 mutation included all patients regardless of subtype of BRAF V600 mutation. 5) Our analysis did not allow for comparison between stage III substages (AJCC v8 IIIA-D). 6) The median follow-up is almost three years, but this may still be insufficient to capture all the events in stage III melanoma.7) Four percent of patients included had melanoma stage IV NED. 8) Finally, we didn’t perform statistical tests for evaluating differences in survival between TT and ICI, but rather performed a descriptive analysis, as the composition of the collectives in the various studies was quite different.

On the other hand, our report has advantages: 1) We included data from 3625 patients receiving adjuvant therapy, 2071 of whom harbored a BRAF mutation. 2) Depending on the country of origin, the patients included in this analysis may have had access to PD-1 based therapies, namely nivolumab, pembrolizumab or the combination of ipilimumab and nivolumab, at the time of relapse, i.e., similar to what is offered nowadays. 3) Grouping of digitalized Kaplan Meier RFS curves by therapeutic strategy showed a high concordance between the survival curves within each therapy in the different publications. 4) We used weighted average calculations which improved the detailed analysis.

In the absence of a clinical trial evaluating face-to-face TT versus ICI in the adjuvant setting, an opportunity to address this question with better statistical quality could be to analyze individual patient data from all patients with BRAFV600 mutation included in the COMBI-AD and Keynote 054 studies, matched by basal staging prognostic factors. This is out of the scope of this manuscript and would require the agreement of at least three different parties (Novartis, EORTC and Merck), but it has been possible before. One recent example, where randomized trials investigating two available therapies will be challenging is that of metastatic uveal melanoma, where an individual rather than aggregated patient analysis has been conducted comparing ipilimumab and nivolumab from the Spanish Multidisciplinary Melanoma Group (GEM 1402) [35] with Tebentafusp (IMCgp100–202) [36,37], unveiling an advantage of tebentafusp over ipilimumab and nivolumab. [38].

Further data is needed, especially on 1) biomarker analysis to identify the patients who would benefit the most from adjuvant therapy, 2) on the identification of patients at risk for developing long-term, irreversible toxicities, and 3) on the potential development of resistance in the adjuvant setting that could impair rescue with systemic therapies in the more advanced, irresectable stages. Finally, longer follow-up is needed to confirm the capture of the majority of recurrences in an adjuvant setting, which depends on a non-standardized schedule of radiological evaluation.

## 5 Conclusion

Here we present the first indirect head-to-head comparison of survival outcomes between adjuvant targeted therapy and immune checkpoint inhibitors in stage III melanoma using real-world data. Our exploratory analysis indicates that targeted therapy may provide longer RFS and DMFS in patients with BRAF V600 mutation, confirming targeted therapy as a beneficial option in this setting.

When deciding on the choice of adjuvant therapy for patients with BRAF mutation in stage III, the following aspects should be taken into consideration when weighing up TT versus ICI: TT shows better RFS data than ICI in the real-world data analysis; TT has a lower toxicity and, in contrast to ICI, it’s not associated with lifelong loss of organ function; clinically meaningful impact in quality of life was found with ICI, but not with TT; and, last but not least, patients may prefer oral to intravenous therapy.

## Supplementary Material

**Appendix A. Supporting information**

Supplementary data associated with this article can be found in the online version at doi:10.1016/j.ejca.2024.115160.

Supplementary Material

## Figures and Tables

**Fig. 2A:**
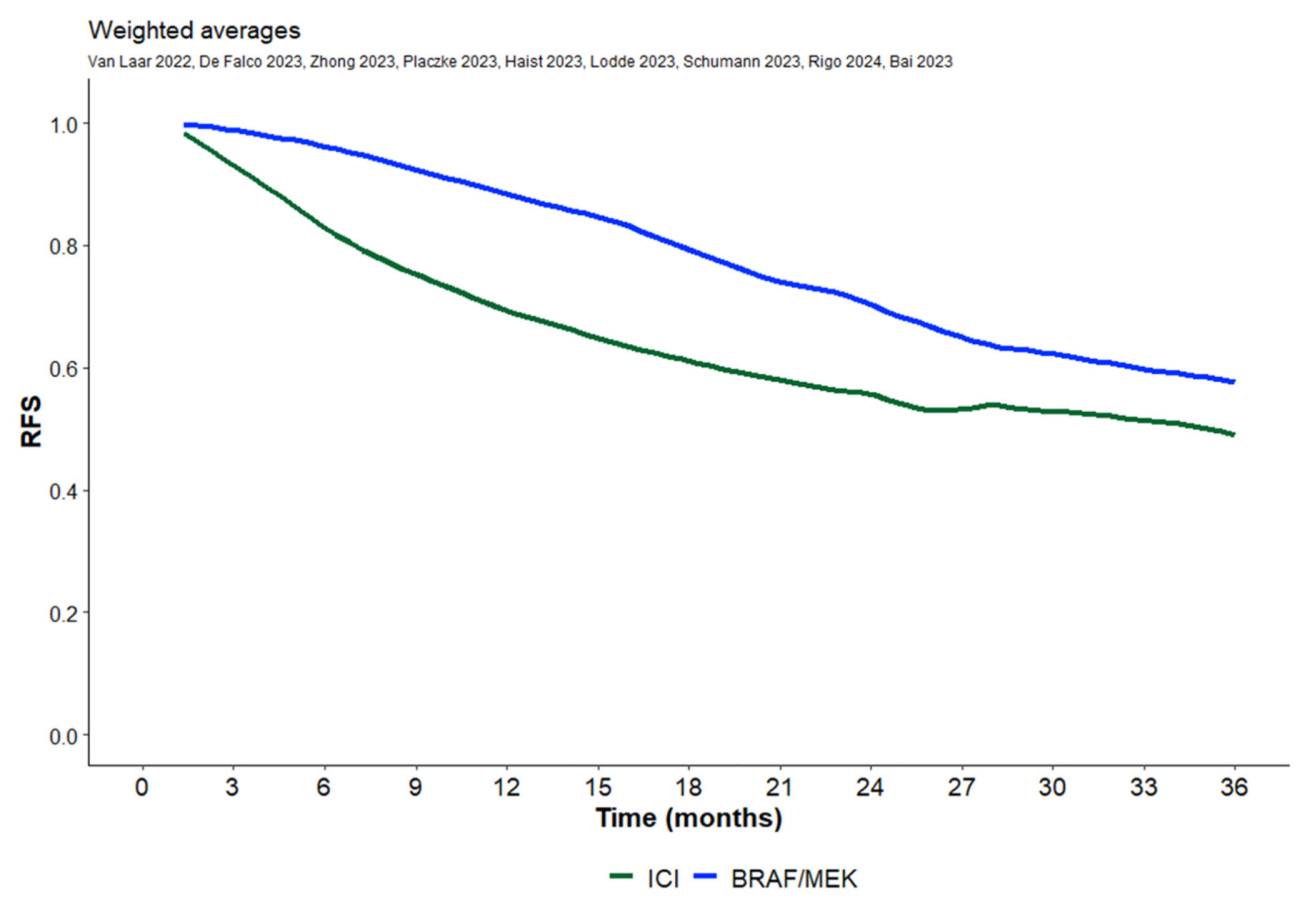
RFS Kaplan Meier curve for immune checkpoint therapy and targeted therapy extracted from 9 publications identified. [8–16] Each publication reported data from more than 65 patients with a BRAF mutation. Total number of patients included is 3625.

**Fig. 2B:**
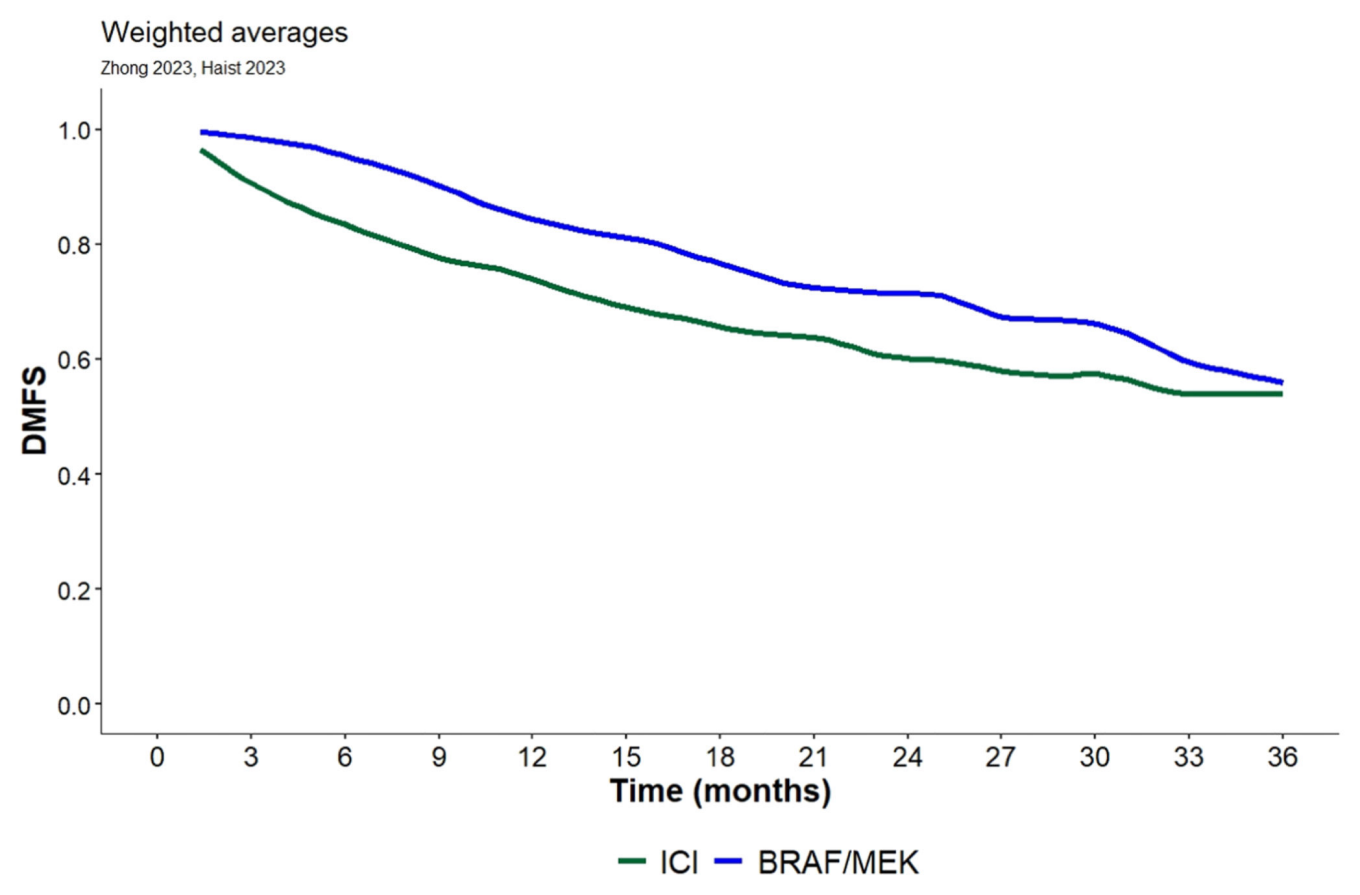
DMFS Kaplan Meier curve for immune checkpoint therapy and targeted therapy extracted from 2 publications [10,12]. Total number of patients included is 608.

**Fig. 2C:**
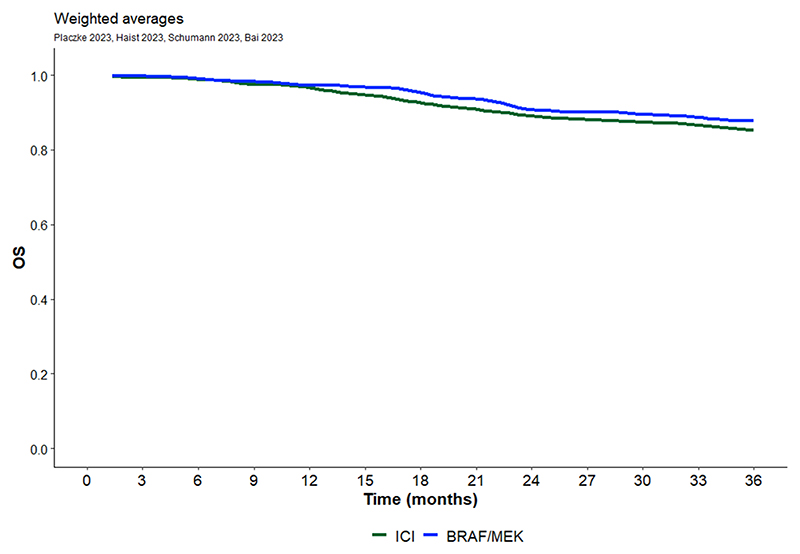
OS Kaplan Meier curve for immune checkpoint therapy and targeted therapy extracted from 4 publications [11,12,14,16]. Total number of patients included is 2559.

**Fig. 3A:**
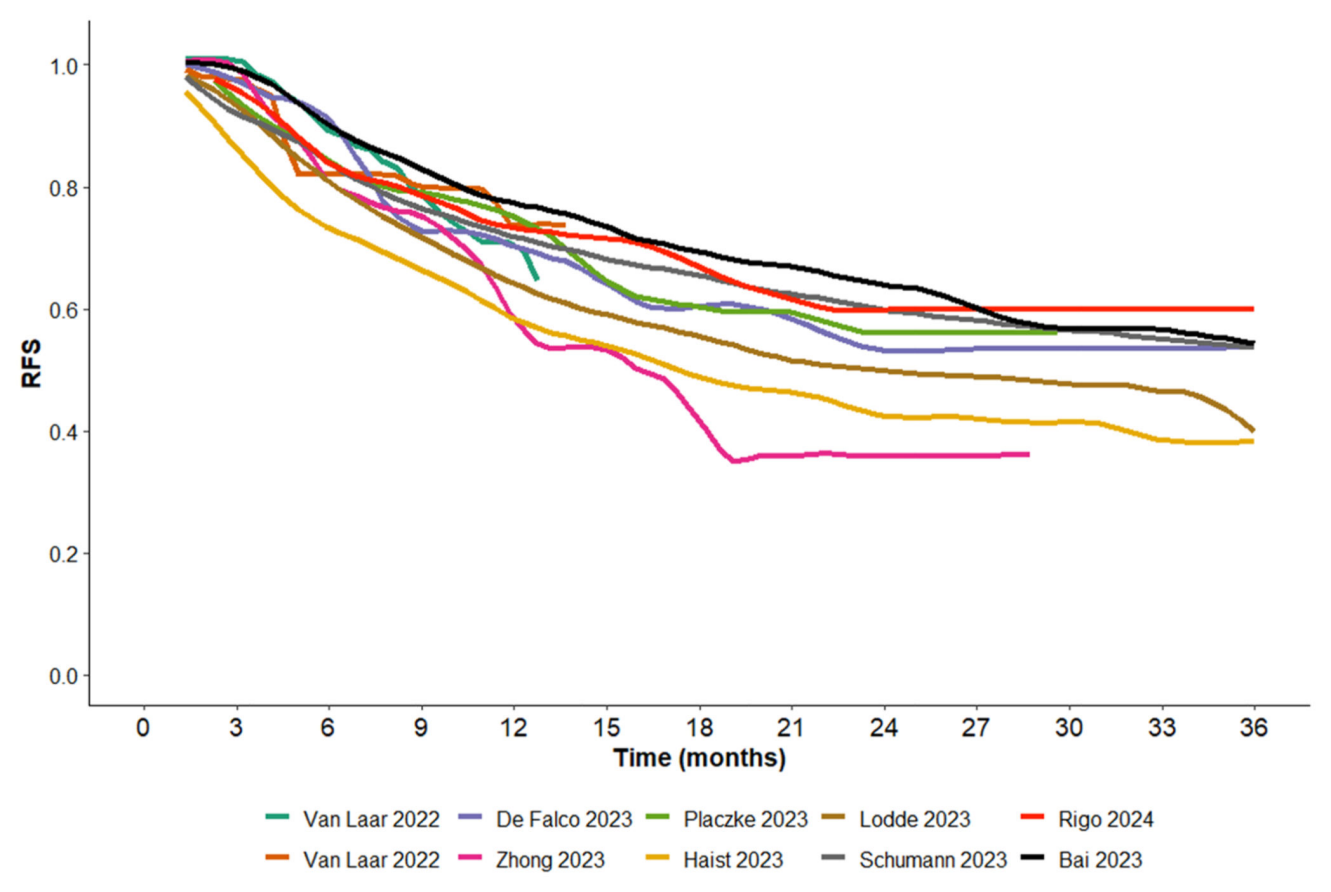
RFS Kaplan Meier curve for immune checkpoint therapy extracted the publications identified below, showing highly concordance among the different publications. The publication from Van Laar 2022 [8] is listed twice in the graphic as Nivolumab and Pembrolizumab were plotted separately in the original publication.

**Fig. 3B:**
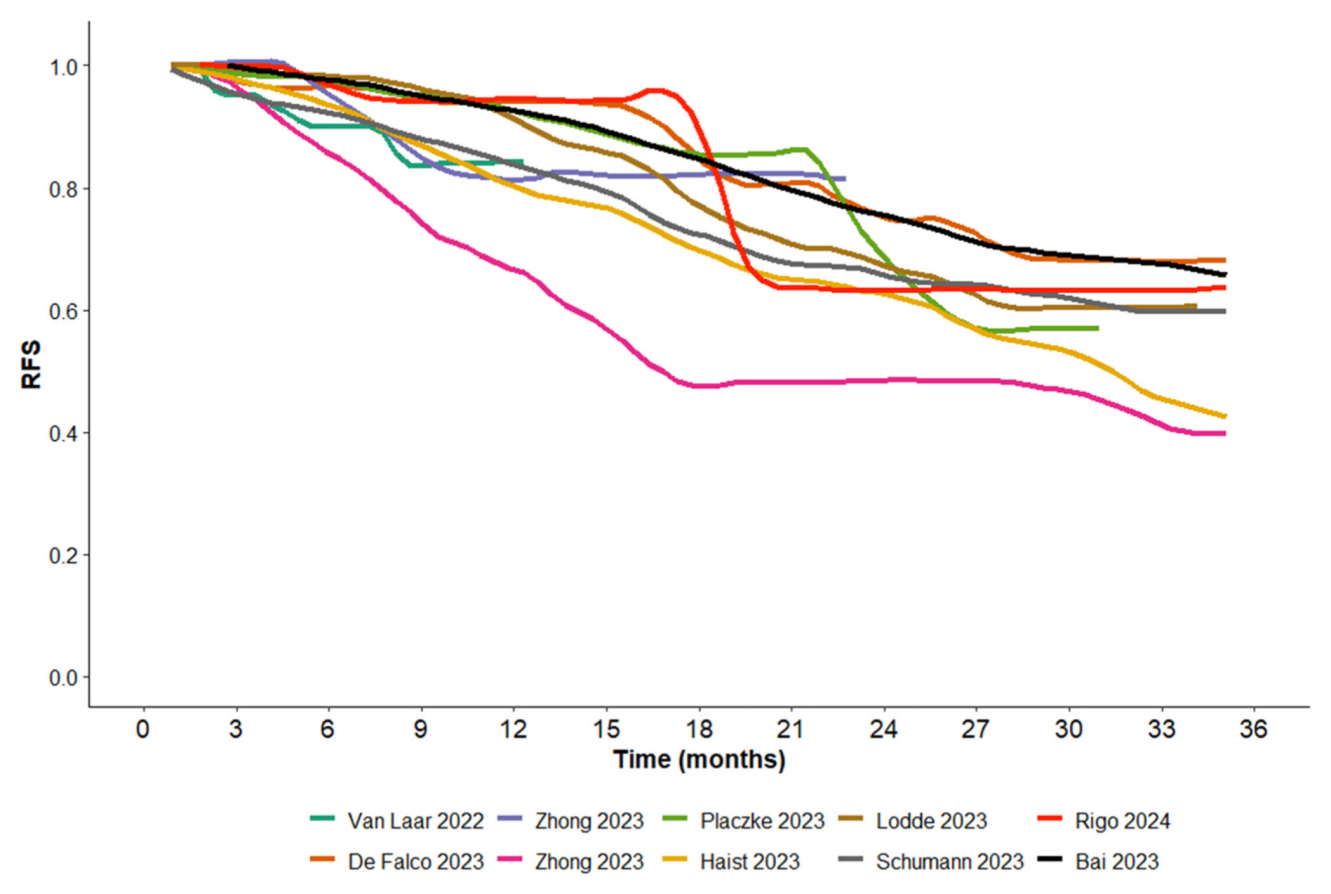
RFS Kaplan Meier curve for targeted therapy extracted the publications identified below, showing highly concordance among the different publications. The publication from Zhong 2023 [10] is listed twice as Vemurafenib and Dabrafenib + trametinib were plotted separately.

**Table 1:** Publications (n = 9) included in the survival analysis.

Publication	Analysis interval	Number of patients included and treated with systemic therapy (TT; ICI)	Number of patients with BRAF mut	FUP	RFS D+T	HR RFS (95 % CI) p value	RFSICI	HR RFS (95 % CI) p value	DMFS D+T	DMFSICI	Comment
van Laar, 2022[8]	01.2019 to 10.2021	122(20/102)	65	Max FUP 13 months	1y RFS 83.0 %	NR	1y RFS nivo 70.3 %, 1y RFS Pembro 72.4 %	NR	NR	NR	20 patients with Stage IV NED included. No information about subtype of V600 mutation
Bai, 2023 [16]	07.2015 to 10.2022	598 (393/205)	598 (83.8 % BRAF V600E; 9,5 % BRAF V600K; 1.3 % other; 5.3 % unknown)	mFUP 33 months (IQR 21–43); TT 29 months [IQR 18–40], PD–1 38 months [IQR 29–50])	51.0 months (95 % CI 41.0-not reached)	Univariate: HR 0.66 (95 % CI 0.50–0.87); P = 0.003; multivariate: HR 0.58, (95 % CI 0.39–0.86); P = 0.007	44.8 months [95 % CI 28.5-NR]	Univariate: HR 0.66 (95 % CI 0.50–0.87) P = 0.003; multivariate: HR 0.58 (95 % CI 0.39–0.86) P = 0.007	NR	NR	OS multivariate, HR 0.90, (95 % CI 0.48–1.70), P = 0.75. 2-yr restricted mean survival time for TT 21.8 months and 19 months for PD–1 (P < 0.001).
De Falco, 2023[9]	Data cut-off for analysis was 31/5/2022	113 (58/55)	75	Total FUP 20.2 months	NR	0.36 (0.16–0.77) p = 0.010	NR	NR	NR	NR	No information about subtype of V600 mutation
Haist 2023 [12]	01.2014 to 07.2022	515 (242/273)	237 (73 % -BRAF V600E; 12.2 % BRAF V600K; 3.3 % BRAF V600D/R)	mFUP 27 months (22.3 to 31.7)	mRFS 11 (8.7 to 13.3)	Adjusted HR 0.52 (95 % CI 0.40 to 0.68) p < 0.001	mRFS 6 months (4.0 to 8.0)		mDMFS 15 months (11.5 to 18.5)	mDMFS 12 months (8.5 to 15.4)	3y OS rate TT 87.4 %;PD–180.5 %
Lodde 2023[13]	06.2018 to 09.2019	589 (110/479)	232 (85 % BRAF V600E; 9 % BRAF V600K; 5 % Other BRAF mutations; 1 % unknonw)	mFUP 25.7 months	2y RFS 67 %	NR	2y RFS 49%	HR 1.99; (95 % CI 1.34–2.96)	NR	NR	2y MSS TT 92 %; PD–1 87 %
Placzke 2023; [11]	02.2019 to 01.2021	248 (101/147)	155	mFUP 13.9 months	2y RFS 65.9 %	NR	2y RFS 56.1 %	NR	2y DMFS 76.5 %	2y DMFS 64.8 %	23 patients with Stage IV NED included.OS TT 87.8 %; PD–1 85.3 %. No information about subtype of V600 mutation
Schumann 2023[14]	01.2017 to 10.2021	1198 (195/1003)	542 (78.8% BRAF V600E; 9,2% BRAF V600K; 12% Other BRAF mutations)	mFUP 17 months	1y RFS 86.5 %; 2y RFS 78.1 %	NR	1y RFS 78.1 %; 2y RFS 67.9	HR 1y 1.998 (95 % CI 1.335–2.991); p = 0.001	NR	NR	105 patients with Stage IV NED included.2y OS TT 95.3 %; PD–1: 93.1 %
Zhong, 2023[10]	01. 2017 to 12.2021	93 (25/25)	93 (100 % BRAF V600E)	mFUP 11months for TT and 22 months for PD–1	mRFS not reached1y RFS 81.7 %; 2y RFS 58.1 %	NR	mRFS 15 months1y RFS 59.0 %; 2y RFS 54.2 %	NR	Not reached	Not recahed	23 patients were treated with Vemurafenib and 20 did not receive adjuvant therapy
Rigo 2024 [15]	09.2017 to 03.2021	149 (20/129)	74 (49.7 % BRAF V600 E or K)	mFUP 22.4 months	mRFS 38.4 %; 1y RFS 94 %; 2y RFS 63 %	NR	mRFS not reached.1y RFS BRAF mut patients 73 %; 2y RFS BRAF mut patients 60 %	NR	NR	NR	7 patients with Stage IV NED included.

HR – Hazard ratio; mFUP – median follow-up; NR – not reported; RFS – relapse free survival; DMFS – distant metastases free survival; MSS – melanoma specific survival; OS – Overall survival; INF – interferon; NA – Not applicable

**Table 2:** RFS, DMFS and OS rates for 6, 12, 24 and 36 months.

	6 months	12 months	24 months	36 months
	%			
**RFS (9 publications)**				
ICI	82.9	69.3	55.5	48.9
TT	95.9	88.2	70.4	57.5
**DMFS (2 publications)**				
ICI	83.4	73.9	60	53.9
TT	95.5	84.4	71.4	55.7
**OS (4 publications)**				
ICI	99.0	96.7	89.2	85.2
TT	99.1	97.0	91.0	87.8
