# Supplementary material for: A comparison of real-world data on adjuvant treatment in patients with stage III BRAF V600 mutated melanoma – Results of systematic literature research
Source: Eur J Cancer. Author manuscript; Available in PMC 2026 Jan 19. (PMC7618644; doi:10.1016/j.ejca.2024.115160)
Supplement: Supplementary Material [file EMS212048-supplement-Supplementary_Material.docx]

Supplementary Material

**Table S1:** Publications retrieved using the search strategy that provided survival data, but were not included in the analysis

| Publication | Analysis interval | Number of patients included and treated with systemic therapy (TT; ICI) | Number of patients with BRAF mut | FUP | RFS D+T | HR RFS (95%CI) p value | RFS  PD-1i | HR RFS (95%CI) p value | DMFS D+T | DMFS  PD-1 | Comment |
| --- | --- | --- | --- | --- | --- | --- | --- | --- | --- | --- | --- |
| Rauwerdink; 2020 [35]** | Started in Jun 2017 | 102 (30; 46) | 39 | Mean FUP 17 months | mRFS 17.9 months | 0.23 (0.07-0.72) p= 0.01 | mRFS 15.3 months | 0.40 (0.16-0.96) p= 0.04 | NR | NR | 7 patients with stage IV NED included. No information about subtype of V600 mutation |
| Amagai 2021 [36]** | 01.2019 to 04.2021 | 36 (36/0) | 36 (86% BRAF V600E; 14% BRAF V600K) | NR | 1y RFS 82.1% | NR | NA | NA | NR | NA | 2 patients with Stage IV NED included. |
| Koelblinger, 2021 [37]** | 12.2017 to 03.2020 | 100 (0;100) | 43 | mFUP 11.5 months (3.0– 28.4 months) | N/A | NR | 12 months estimates 64.8 % | NR | NR | 12 months estimates 77.4 % | 8 patients with stage IV NED included. No information about subtype of V600 mutation |
| Hoffmann, 2022 [38]** | 01.2018 to 16.06.2021 | 109 (3/90) | 44 (91% BRAF V600E/K; 9 % BRAF mutation unknown) | mFUP 11.3 months | NR | NR | 1y RFS Nivo 77.1%, 1y RFS Pembro 73.5% | NR | NR | NR | 5 patients with stage IV NED included. |
| Sun 2022[39]** | 01.2017 to 12.2021 | 174 (0/115) | 41 | mFUP 21 months | NA | NA | mDFS 22 months | NR | NR | NR | 22 patients received INF and 37 patients did not receive adjuvant therapy. No information about subtype of V600 mutation |
| Li, 2023 [40]** | 2017 to 2021 | 199 (0/126) | 23 | mFUP 21 months  for patients treated with PD-1 | NR | NR | mRFS 23 months  1y RFS 59.2%, 2y RFS 49.4% | NR | NR | 1y DMFS 84.7%; 2y DMFS 69.4% | Patients treated with PD-1 monotherapy, INF or only observation were evaluated. 136 patients with acral melanoma were included.  No information about subtype of V600 mutation |
| Manzano 2023 [41]# | 10.2020 to 03.2021 | 65 (65/0) | 65 | mFUP 20 months | 1y RFS 95.3%; 2y RFS 72.9%; mRFS not reached | NR | NA | NA |  |  | OS 1y, 2y, and 3y were 100%; 90.6%; and 83.2% respectively.  2 patients with Stage IV NED included. No information about subtype of V600 mutation |
| Rogiers, 2023 [29]** | 01.2029-01-2021 | 152 (0/152) | 58 | mFUP 18.5 months | NA | NA | 1y RFS 74.7%, 18 months RFS 68.4% | NR | NA | NR | 17 patients with Stage IV NED included.  No information about subtype of V600 mutation |
| Ascierto 2023; [42]# | 11.2018 to 06.2019 | 611 (0/611) | 157 | mFUP 23 months | NA | NA | 1y RFS 76.6% ; 2y RFS 59.6%; BRAF mut patients 1y RFS 83.8%; 2y RFS: 65.6% |  | 1y DMFS 83.7%, 2y DMFS 71.2% ; BRAF mut patients 2y RFS 79.4% |  | 1y OS 93.8%; 2y OS 85.5%  141 patients  with stage IV NED included.  No information about subtype of V600 mutation |
| Grover 2023 [43]$ | 01.2018 to 07.2021 | 628 (80/256) | 167 | mFUP 31 months | 2y RFS 98.6% | NR | 2y RFS all patients 79.3%.  2y RFS BRAF mut patients 71.6% | NR | 2y DMFS 100% | 2y DMFS all patients 88.4%.  2y DMFS BRAF mut patients 83.5% | Only stage IIIA patients were included.  292 patients did not receive adjuvant therapy.  No information about subtype of V600 mutation |
| Holmstroem 2024 [44]# | 11.2018 to 01.2022 | 785 (0/785) | 234 | mFUP 25.6 months | NA | NA | mRFS not reached; 3y RFS 59% | NR | NA | NR | Stage III/IV  3y MSS rate 87.4%.  105 patients with Stage IV NED. No information about subtype of V600 mutation |

**Exclusion criteria for the survival curves**

**- Included less than 65 patients with BRAFV600 mutation; # - Only Treated with ICI or TT; $ - Only stage III.

HR – Hazard ratio; NED – no evidence of disease; mFUP – median follow-up; NR – not reported; RFS – relapse free survival; DMFS – distant metastases free survival; MSS – melanoma specific survival; OS – Overall survival; INF – interferon; NA – Not applicable
